# Enzyme-based lignocellulose hydrolyzation – Brief data survey for cellulase performance characterization on behalf of the Sauter mean diameter of raw material particles

**DOI:** 10.1016/j.dib.2015.11.008

**Published:** 2015-11-18

**Authors:** Robert Glaser

**Affiliations:** Leibniz Institute for Agricultural Engineering Potsdam-Bornim, Max-Eyth-Allee 100, 14469 Potsdam, Germany

**Keywords:** Lignocellulose, Inhomogeneous substrate, Cellulase, Sauter mean diameter, Empirical model

## Abstract

The data presented here supports the informational background of enzyme-based lignocellulose hydrolyzation, cellulase characterization, and sugar yield prediction for the work “Enzyme-based lignocellulose hydrolyzation – Sauter mean diameter of raw materials as a basis for cellulase performance characterization and yield prediction” by Glaser [1]. Glucose yields from the enzymatic hydrolysis of the raw materials were shown as a function of cellulase enzyme loading as well as of particle size with different solid loading. The data for the proposed methods of the determination of enzyme activity in inhomogeneous samples of lignocellulosic raw materials are presented. The data of the empirical model that was developed for the prediction of hydrolysis yields for different enzyme concentrations, substrate specific particle size, and solid loadings, are given. Data are also given in relation of terms of scale-up opportunities.

**Specifications Table**

**Table:** 

Subject area	Biotechnology
More specific subject area	Bioresources, cellulases
Type of data	Table
How data was acquired	– Enzymatic hydrolyses of lignocellulosic raw materials – Glucose determination by high-pressure liquid chromatography – Mathematical model equation, parameter adjustment, and model simulation – Torque measurement by the used Heidolph stirrer and determination of the power number of the used setup
Data format	Raw, filtered, analyzed
Experimental factors	The raw materials were milled and used naturally dried. Different amounts of protein of the cellulase enzyme mixtures were used for hydrolyses of the lignocellulose raw materials. Characterization of cellulase enzyme mixtures by different particle size fractions of wheat straw was undertaken. Hydrolysis data are used to define a kinetic unit for the estimation of cellulase performance in inhomogeneous raw materials. The defined kinetic unit was used for the yield prediction with an empirically defined model equation in different scales
Experimental features	Using Sauter mean diameter of lignocellulosic raw materials for cellulase characterization and yield prediction in scale-up processes
Data source location	Potsdam, Brandenburg, Germany
Data accessibility	Data is presented in this article

## 1 Value of data

• The estimation of cellulase performance for industrial-scale processes holds special challenges. There exists a gap between the enzyme performance in a laboratory and in large-scale processes. As a standard tool for cellulase characterization, the determination of the filter paper units (FPU) through the filter paper assay (FPA) [2] is given.
• With the data which is given in the following and the methods given by Glaser [1], it is possible to define a self-specified cellulase unit via easy measurable process properties, e.g. cellulase enzyme loading and mixture, lignocellulose solid loading, type of lignocellulose, and particle size distribution of the given raw materials. The thereby defined cellulase unit will be facile and generally understandable.
• The data given here will provide a first step to characterize and compare a user-defined process due to the easy application of the model [1].

## 2 Data

The cellulase performance was determined as a function of cellulase enzyme loading, particle sizes of different raw materials, and different solid loadings.

Raw materials such as wheat straw, pulverized wheat straw, grass, pine wood, aspen wood, and rice straw as well as glucose yields were used.

Torque data and power numbers of the reactor setting were obtained for scale comparison.

### 2.1 Lignocellulose raw material characterization by Sauter mean diameter

Different kinds of lignocellulose raw materials were used in the work by Glaser [1]. The raw materials used there were wheat straw (WS) with a Sauter mean diameter (SMD) of 435 µm, pulverized wheat straw (pWS) with an SMD of 202 µm, aspen wood with an SMD of 513 µm, pine wood (PW) with an SMD of 483 µm, grass with an SMD of 153 µm, and rice straw (RS) with an SMD of 169 µm. In Table 1 the data of the particle size distribution which was used to determine the SMD are shown.

### 2.2 Cellulase performance characterization

Five fraction classes with a range of different particle sizes were used for evaluation of cellulase performance on the wheat straw. Therefore, four different protein amounts of the cellulase mixtures were used. The used types of cellulases such as CTec2 (CT2), HTec2 (HT2), and the cellulase mixtures provided by the Moscow State University, are described in a more detailed way in the main article [1]. Data of hydrolyses as mean values of the glucose yield are shown in Table 2. The glucose yield differs depending on the cellulase amount used in the experiment. To smooth the data for further use, an exponential equation was adjusted to the data by fitting two parameters (see Eq. (2) in Section 3.1). The data of the fitted parameters are shown in Table 3. The kinetic unit, which was proclaimed on the basis of the cellulase performance, depends on the cellulase enzyme loading and the mean particle size of the wheat straw fraction. Further details and descriptions of the determination of the wheat straw units (WSU) are given in the main article [1].

### 2.3 Hydrolyses in shaking flask and 2 L and 10 L scale stirred bioreactors

The defined WSU was used to predict hydrolyses yield of the sugars from the different given raw materials in differently scaled shacked and stirred bioreactors. For these hydrolyses, different amounts of protein were used. Data of hydrolyses done in shaking flasks are shown in Table 4 for different lignocellulose raw materials in combination with the used WSU [1]. Data of hydrolyses of WS, PW, and AW in stirred bioreactors of 2 L and 10 L scale are shown in Table 5 alongside the used WSU [1]. The given data provide the opportunity to compare the glucose yield kinetic between different cellulase mixtures and raw materials.

### 2.4 Torque and power numbers of Ruston turbine equipment

For the scale-up from the shacked reaction tubes over shacked flasks towards the 2 L and 10 L bioreactor vessels, the power number was estimated by torque measurement. Table 6 shows the torque data of the 2 L vessel with the single and double used Rushton turbines. Table 7 shows the data for the 10 L vessel. The stable power number was derived in turbulent flow. Different scale-up criteria can be assumed, for example, constant power input and constant flow characteristics described by a constant Reynolds number. However, as a scale criterion between the 2 L scale and the 10 L scale, a constant stirring speed was used. See Glaser [1] for more details. For more information concerning the determination of the power characteristics, refer Kraume [3], Sieblist et al. [4], and Zlokarnik [5].

The Leibniz-Institute of Agricultural Engineering is interested and open for new interdisciplinary cooperation, especially in biotechnology in the area of bioeconomy. It provides a working environment with enthusiastic researchers. With its pilot plant bio-refinery, it is also interested in topics of biotechnological production of value added products, such as lactic acid or succinic acid from industrial residues and wastes. The Leibniz-Institute of Agricultural Engineering defines principles for best practice of scale-up and scale-down of diverse biotechnological processes.

## 3 Experimental design, materials, and methods

To check for statistical significance, the data were evaluated by the analysis of variance (ANOVA) in combination with variances and standard mean deviations in correlation coefficients. Those data were given in the main article [1].

### 3.1 Characterization of lignocellulose raw materials by Sauter mean diameter

The Sauter mean diameter was calculated through the mean fraction diameter of a particle class *d*_*m*,*i*_ and the mass fraction of the particle class *m*_3,*i*_ (Eq. (1)). The fractions of the particle classes were determined by sieving with standard screens and different mesh sizes.

(1) $$\mathrm{SMD}=\frac{1}{\sum_{i=1}^{N}\frac{\mu_{m,i}}{d_{m,i}}}$$

### 3.2 Hydrolyses in reaction tubes for cellulase characterization

The amount of 200 mg of WS was weighed out directly in reaction tubes and charged with two milliliters of 50 mM sodium acetate buffer with pH 5 and preheated to 52 °C, as were the enzymes. Two milliliters of enzyme dilutions were added to the WS-filled tubes. Resulting protein concentrations of cellulase enzyme solution were 1.25, 2.5, 5, and 10 mg/mL. The closed tubes were then placed on a planar shaker at 100 rpm in combination with an incubator at 52 °C for 60 min (Certomat U, B. Braun Diessel Biotech.). To stop the hydrolysis, the tubes were placed in a boiling water bath for 20 min. Afterwards they were cooled down to −20 °C and stored for further use.

A regression fitting with an exponential equation (Eq. (2)) was done in order to smooth the data of the hydrolysis in the reaction tubes for the definition of a kinetic unit [1]. The fitted parameters are shown in Table 3. The parameter *S* [g] was defined by the amount of the cellulose part of the lignocellulosic substrate, while *C* [–] was defined as the conversion factor of the substrate after the hydrolysis. The parameter *k* [1/*g_p_*] defines the turnover of cellulose to glucose per gram of cellulase protein in 60 min while *E*_*p*_ is defined by the amount of the used cellulase protein [*g_p_*]. The parameters *C* and *k* were the optimized parameters by minimizing the root mean squares between the experimental data and data given by Eq. (2).

(2) $$Y=S∙C∙(1-\exp\left(-k∙E_{p}\right)$$

### 3.3 Technical-scale hydrolyses in shaking flask, 2 L and 10 L stirred bioreactors

Shaking flask hydrolyses experiments were carried out in Erlenmeyer flasks charged with 200 mL of 50 mM sodium acetate buffer at pH 5, 52°C, and 5% w/v of solid loading. The flasks were shaken at 100 rpm. In comparison to this shaking flask system, hydrolyses were also carried out in 2 L and 10 L stirred bioreactors. The WS hydrolyses took place with 5% w/v and 7.5% w/v solid loadings, and the AW and PW were used at 5% w/v solid loading. The working volumes were 1 L for the 5% w/v or 1.5 L and 8 L for hydrolyses at 7.5% w/v of solid loadings.

### 3.4 Detection of sugars

The detection of sugars was done by anion-exclusion high-pressure liquid chromatography (HPLC) with a EurokatH column (300 mm×8 mm, 10 lm, eluent: 0.01 N H_2_SO_4_, RI 75 detector) (KNAUER). The column was used at a constant temperature of 35 °C and under constant acidic pH conditions with 0.005 mol/L H_2_SO_4_ in the mobile phase. The injection volume was 10 µL at a pressure of 1.5 MPa.

### 3.5 Determination of torque and power numbers

The experimental determination of the power input (*P*_*effective*_ [W]) was accomplished using the internal torque sensor of the used Heidolph stirrer (RZ 2052). The power input into the loaded stirred vessel of the bioreactor (*P*_*loaded*_ [W]) must be subtracted by the power input into the unloaded vessel of the bioreactor (*P*_*drained*_ [W]). Due to this setup, the friction and other losses of the agitation system were considered [4].

(3) $$P_{\mathrm{effective}}=P_{\mathrm{loaded}}-P_{\mathrm{drained}}$$

The data of effective power input were used for the calculation of the corresponding power number of the stirring system by the following Eq. (4).

(4) $$\mathrm{Po}=\frac{P_{\mathrm{effective}}}{\rho_{L}∙n^{3}∙d^{5}}=\frac{2∙\pi ∙n∙\left(M_{\mathrm{loaded}}-M_{\mathrm{drained}}\right)}{\rho_{L}∙n^{3}∙d^{5}}$$

where *ρ*_*L*_ [kg/m^3^] is the liquid density, *n* [s^−1^] is the stirrer speed, and *M* [N m] is the measured torque. *M*_*loaded*_ is the torque of the loaded and stirred vessel while *M*_*drained*_ is the torque of the unloaded stirred bioreactor vessel.

The 2 L reactor was equipped with two six-blade Rushton turbines and the physical parameters were: the mean stirrer diameter: *d*=0.053±0.001 m, the relation of the stirrer diameter and the inner vessel diameter: *d*/*D*=0.42, the relation of the lower Rushton turbine and vessel diameter: *h*_1_/*D*=0.08, the relation of the upper Rushton turbine and vessel diameter: *h*_2_/*D*=0.32, and the relation of the filling height and vessel diameter: *H*_*fill*_/*D*=0.71 at 300 rpm. The reactor of the 10 L scale was also equipped with two six-blade Rushton turbines with parameters of *d*=0.07 m, *d*/*D*=0.42, *h*_1_/*D*=0.71, *h*_2_/*D*=1.05, and *H*_*fill*_/*D*=1.58 at 300 rpm.

## Figures and Tables

**Table 1:** Particle size distribution of milled wheat straw, pulverized wheat straw, pine wood, aspen wood, rice straw, and grass.

*d*_*m*,*i*_ [µm]	WS	pWS	AW	PW	Grass	RS
900	6.31	0.04	42.40	36.05	0.07	0.00
715	35.83	0.08	24.08	28.02	0.22	0.02
472.5	42.56	13.08	19.94	20.82	2.55	9.83
282.5	4.19	27.86	5.73	6.47	3.28	13.99
225	3.40	31.76	2.85	2.87	28.14	25.75
162.5	6.61	20.48	4.03	3.82	44.22	34.58
107.5	0.84	3.91	0.46	0.52	15.93	10.59
85	0.14	0.69	0.16	0.40	3.67	2.90
75.5	0.03	0.30	0.11	0.71	0.62	0.55
67	0.05	0.35	0.14	0.30	0.24	0.28
31.5	0.03	1.46	0.10	0.02	1.05	1.52
SMD [µm]	435	202	513	483	153	169

**Table 2:** Mean glucose yields of two 60 min hydrolyses of different particle classes of WS in shacked reaction tubes with different enzyme loadings.

PS	800–630 µm		630–315 µm		315–250 µm		250–200 µm		200–125 µm		125 µm and lower	
Protein	Glucose yield											
[mg/mL]	[g]	[%]	[g]	[%]	[g]	[%]	[g]	[%]	[g]	[%]	[g]	[%]

R1												
10	0.00399	3.80	0.01303	12.3	0.00802	7.60	0.01023	9.70	0.01475	14.0	0.03129	29.6
5	0.00339	3.2	0.01296	12.3	0.00742	7.0	0.00929	8.8	0.01204	11.4	0.02705	25.6
2.5	0.00268	2.5	0.00914	8.6	0.00552	5.2	0.00732	6.9	0.00951	9.0	0.02407	22.8
1.25	0.00245	2.3	0.00769	7.3	0.00393	3.7	0.00468	4.4	0.00675	6.4	0.01845	17.5
R2												
10	0.00317	3.0	0.01019	9.6	0.00634	6.0	0.00791	7.5	0.01208	11.4	0.02937	27.8
5	0.00290	2.7	0.00837	7.9	0.00540	5.1	0.00733	6.9	0.00962	9.1	0.02691	25.5
2.5	0.00176	1.7	0.00636	6.0	0.00354	3.4	0.00624	5.9	0.00796	7.5	0.01954	18.5
1.25	0.00118	1.1	0.00495	4.7	0.00227	2.2	0.00332	3.1	0.00416	3.9	0.01332	12.6
R3												
10	0.00424	4.0	0.01189	11.2	0.00788	7.4	0.01096	10.4	0.01443	13.6	0.02964	28.0
5	0.00311	2.9	0.00977	9.2	0.00636	6.0	0.00962	9.1	0.01140	10.8	0.02597	24.6
2.5	0.00235	2.2	0.00807	7.6	0.00545	5.2	0.00810	7.7	0.00857	8.1	0.02251	21.3
1.25	0.00208	2.0	0.00672	6.4	0.00398	3.8	0.00525	5.0	0.00617	5.8	0.01711	16.2
R4												
10	0.00260	2.5	0.01101	10.4	0.00616	5.8	0.00917	8.7	0.01275	12.1	0.03109	29.4
5	0.00228	2.2	0.00949	9.0	0.00517	4.9	0.00762	7.2	0.01035	9.8	0.02445	23.1
2.5	0.00201	1.9	0.00774	7.3	0.00419	4.0	0.00602	5.7	0.00781	7.4	0.02112	20.0
1.25	0.00175	1.7	0.00599	5.7	0.00321	3.0	0.00443	4.2	0.00527	5.0	0.01779	16.8
CT2												
10	0.00425	4.0	0.03396	32.1	0.00880	8.3	0.01666	15.8	0.01497	14.2	0.03953	37.4
5	0.00425	4.0	0.02317	21.9	0.00880	8.3	0.01666	15.8	0.01497	14.2	0.03953	37.4
2.5	0.00425	4.0	0.02347	22.2	0.00880	8.3	0.01666	15.8	0.01497	14.2	0.03953	37.4
1.25	0.00224	2.1	0.01480	14.0	0.00564	5.3	0.00948	9.0	0.01049	9.9	0.02957	28.0

**Table 3:** Adjusted parameters k and C of Eq. (2).

PS	800–630 µm	630–315 µm	315–250 µm	250–200 µm	200–125 µm	<125 µm

R1						
k [1/*g_E_*]	105.98	121.53	124.63	122.65	109.91	178.40
C [*g_S_*]	0.0377	0.1276	0.0761	0.0971	0.1364	0.2804
R2						
k [1/*g_E_*]	84.306	112.07	125.00	126.97	91.514	112.95
C [*g_S_*]	0.0317	0.0939	0.0550	0.0759	0.1151	0.2813
R3						
k [1/*g_E_*]	134.33	166.13	124.63	132.70	94.149	168.98
C [*g_S_*]	0.0402	0.1126	0.0750	0.1020	0.1354	0.2682
R4						
k [1/*g_E_*]	223.31	144.60	124.63	118.44	95.515	156.22
C [*g_S_*]	0.0229	0.1001	0.0569	0.0843	0.1205	0.2700
CT2						
k [1/*g_E_*]	184.45	113.98	124.63	135.93	254.47	283.62
C [*g_S_*]	0.0402	0.3212	0.0832	0.2027	0.1443	0.3739

**Table 4:** Glucose yield of pWS, RS, grass, and PW hydrolyses done in 200 mL shaking flask.

	Pulverized wheat straw			Rice straw			Grass			Pine wood		
T	R1R2	R3R4	CT2	R1R2	R3R4	CT2	R1R2	R3R4	CT2	R1R2	R3R4	CT2

0 h	0.000	0.000	0.000	0.297	0.226	0.319	0.254	0.268	0.264	0.000	0.000	0.000
3 h	0.000	0.000	0.000	0.817	0.745	1.008	0.547	0.598	0.581	0.086	0.039	0.179
6 h	0.107	0.091	0.168	0.831	0.684	1.006	0.539	0.633	0.567	0.598	0.535	0.476
12 h	0.138	0.100	0.174	0.905	0.728	0.972	0.593	0.661	0.661	0.725	0.655	0.547
24 h	0.200^a^	0.164^a^	0.293^a^	0.875	0.809	1.011	0.598	0.672	0.714	0.893	0.781	0.645
48 h	0.246	0.205	0.384	0.943	0.768	0.981	0.576^b^	0.683^b^	0.657^b^	0.959	0.800	0.764
WSU	100	95	48	137	128	65	122	115	58	37	36	18

T: hydrolysis time.

a Hydrolysis time 26 h.

b Hydrolysis time 48.5 h.

**Table 5:** Hydrolyses data in 2 L and 10 L stirred bioreactor.

WS 5%; 2 L					WS 7.5%; 2 L				WS 7.5%; 10 L			PW 5%; 2 L					AW 5%; 2 L		
T	R1R2R3R4		T	CT2	T	R1R2	R3R4	CT2	T	R1	R3	T	R1R2R3R4		T	CT2	T	R1R2R3R4	

0 h	0.00	0.00	0 h	0.00	0 h	0.00	0.00	0.00	0 h	0.00	0.00	0 h	0.00	0.00	0	0.00	0	0.00	0.00
3 h	8.68	8.48	1 h	6.14	3 h	26.54	24.97	20.00	0.5 h	2.59	2.27	3 h	2.09	1.50	3	0.80	3	8.97	7.90
6 h	10.1	9.18	3 h	10.54	6 h	29.16	28.23	25.13	1.5 h	5.51	4.21	17 h	3.86	3.54	6.3	2.20	6	12.3	10.3
24 h	11.4	10.0	6 h	12.28	12 h	30.66	28.90	29.10	2.5 h	7.02	5.96	25 h	4.28	3.99	12	2.59	18	18.1	16.0
29 h	10.9	10.2	24 h	14.03	24 h	33.39	30.22	31.31	6 h	10.7	9.89	42 h	4.40	4.15	26	3.06	27.3	20.8	18.7
46 h	11.7	10.3	30 h	14.36					22 h	11.4	10.1	64 h	4.15	4.34	50	3.92	53.3	23.3	22.2
WSU	68	61		39		137	118	77		67	65		90	62		55		273	237

T: Hydrolysis time.

**Table 6:** Torque and power numbers of Ruston turbine equipment of 2 L stirred bioreactors.

Rpm	300	400	500	600	700	800	900	1000


Torque of the drained reactor vessel								
Measurement #1	6.40	6.60	6.80	7.20	7.40	7.70	8.20	9.10
Measurement #2	6.30	6.50	6.80	7.30	7.30	7.70	8.50	9.00
Measurement #3	6.30	6.40	6.90	7.10	7.40	7.70	8.30	8.90
Mean *M*_*drained*_	6.33	6.50	6.83	7.20	7.37	7.70	8.33	9.00

Torque of the lower Rushton-turbine								
Measurement #1	6.40	7.30	7.70	8.60	9.30	10.50	11.80	13.40
Measurement #2	6.40	7.20	7.80	8.50	9.20	10.40	11.90	13.30
Measurement #3	6.40	7.20	7.90	8.60	9.30	10.30	11.70	13.50
Mean *M*_*loaded*_	6.40	7.23	7.80	8.57	9.27	10.40	11.80	13.40
*M*_*effective*_	0.07	0.73	0.97	1.37	1.90	2.70	3.47	4.40
*Po*	0.91	5.63	4.75	4.66	4.76	5.18	5.26	5.40
Mean *Po*	5.09							

Torque of the upper Rushton-turbine								
Measurement #1	6.40	7.10	7.70	8.30	9.10	10.10	11.40	13.00
Measurement #2	6.40	7.20	7.60	8.40	9.30	10.20	11.50	13.10
Measurement #3	6.50	7.10	7.60	8.40	9.10	10.00	11.50	12.90
Mean *M*_*loaded*_	6.43	7.13	7.63	8.37	9.17	10.10	11.47	13.00
*M*_*effective*_	0.10	0.63	0.80	1.17	1.80	2.40	3.13	4.00
*Po*	1.36	4.86	3.93	3.98	4.51	4.61	4.75	4.91
Mean *Po*	4.51							

Combined torque of the lower and upper Rushton-turbine								
Measurement #1	6.70	7.60	8.30	9.00	10.10	11.00	12.50	14.20
Measurement #2	6.80	7.50	8.50	9.10	10.00	11.30	12.40	14.10
Measurement #3	6.80	7.30	8.20	9.00	9.70	11.10	12.60	14.30
Mean *M*_*loaded*_	6.77	7.47	8.33	9.03	9.93	11.13	12.50	14.20
*M*_*effective*_	0.43	0.97	1.50	1.83	2.57	3.43	4.17	5.20
*Po*	5.91	7.42	7.37	6.25	6.43	6.59	6.32	6.39
Mean *Po*	6.68							

**Table 7:** Torque and power numbers of Ruston turbine equipment of 10 L stirred bioreactors.

Rpm	100	200	300	350	400	450	500	550	600	650


Torque of the drained reactor vessel										
Measurement #1	5.10	6.00	6.30	6.35	6.40	6.55	6.70	6.85	7.00	7.15
Measurement #2	5.00	5.70	6.30	6.35	6.40	6.50	6.60	6.70	6.80	6.90
Measurement #3	5.00	5.80	6.20	6.25	6.30	6.55	6.80	7.05	7.30	7.55
Mean *M*_*drained*_	5.03	5.83	6.27	6.32	6.37	6.53	6.70	6.87	7.03	7.20

Torque of the lower Rushton-turbine										
Measurement #1	5.10	6.50	8.40	9.40	10.60	11.80	13.40	15.10	17.00	19.20
Measurement #2	5.20	6.60	8.30	9.50	10.50	11.70	13.50	15.00	16.70	18.70
Measurement #3	5.10	6.50	8.50	9.30	10.60	12.00	13.20	15.20	17.20	18.00
Mean *M*_*loaded*_	5.13	6.53	8.40	9.40	10.57	11.83	13.37	15.10	16.97	18.63
*M*_*effective*_	0.10	0.70	2.13	3.08	4.20	5.30	6.67	8.23	9.93	11.43
*Po*	1.35	2.36	3.20	3.39	3.54	3.53	3.60	3.67	3.72	3.65
Mean *Po*	3.62									

Torque of the upper Rushton-turbine										
Measurement #1	5.30	6.80	8.90	10.20	11.50	12.90	14.90	17.00	19.30	21.90
Measurement #2	5.10	6.70	9.00	10.10	11.40	12.80	14.80	16.90	19.40	21.70
Measurement #3	5.20	6.90	8.80	10.00	11.60	13.00	15.10	16.80	19.50	21.80
Mean *M*_*loaded*_	5.20	6.80	8.90	10.10	11.50	12.90	14.93	16.90	19.40	21.80
*M*_*effective*_	0.17	0.97	2.63	3.78	5.13	6.37	8.23	10.03	12.37	14.60
*Po*	2.25	3.26	3.94	4.16	4.33	4.24	4.44	4.47	4.63	4.66
Mean *Po*	4.46									

Combined torque of the lower and upper Rushton-turbine										
Measurement #1	5.30	7.80	11.00	12.30	14.90	17.20	20.30	24.00		
Measurement #2	5.50	7.60	10.90	12.50	14.80	17.60	20.50	24.50		
Measurement #3	5.40	7.70	11.00	12.20	15.10	17.30	20.80	24.00		
Mean *M*_*loaded*_	5.40	7.70	10.97	12.33	14.93	17.37	20.53	24.17		
*M*_*effective*_	0.37	1.87	4.70	6.02	8.57	10.83	13.83	17.30		
*Po*	4.94	6.29	7.04	6.62	7.22	7.21	7.46	7.71		
Mean *Po*	7.21									
